# Coffee Intake and Obesity: A Meta-Analysis

**DOI:** 10.3390/nu11061274

**Published:** 2019-06-05

**Authors:** Ariel Lee, Woobin Lim, Seoyeon Kim, Hayeong Khil, Eugene Cheon, Soobin An, SungEun Hong, Dong Hoon Lee, Seok-Seong Kang, Hannah Oh, NaNa Keum, Chung-Cheng Hsieh

**Affiliations:** 1 Dartmouth College, Hanover, NH 03755, USA; ariel.lee.21@dartmouth.edu; 2 Department of Food Science and Biotechnology, Dongguk University, Goyang 10325, Korea; dladnqls1234@naver.com (W.L.); sy98727@naver.com (S.K.); kyk3079@naver.com (H.K.); eugenecheon9@naver.com (E.C.); asv0321@naver.com (S.A.); olivialol@naver.com (S.H.); sskang@dongguk.edu (S.-S.K.); 3 Department of Nutrition, Harvard T.H. Chan School of Public Health, Boston, MA 02138, USA; dhlee@mail.harvard.edu; 4 College of Health Sciences, Korea University, Seoul 02841, Korea; 5 Department of Epidemiology, Harvard T.H. Chan School of Public Health, Boston, MA 02138, USA; CHESIEH@hsph.harvard.edu

**Keywords:** coffee intake, obesity, adiposity, body mass index, waist circumference, meta-analysis

## Abstract

Many studies have explored the relationship between coffee—one of the most commonly consumed beverages today—and obesity. Despite inconsistent results, the relationship has not been systematically summarized. Thus, we conducted a meta-analysis by compiling data from 12 epidemiologic studies identified from PubMed and Embase through February 2019. The included studies assessed obesity by body mass index (BMI, a measure of overall adiposity) or waist circumference (WC, a measure of central adiposity); analyzed the measure as a continuous outcome or binary outcome. Using random effects model, weighted mean difference (WMD) and 95% confidence interval (CI) were obtained for continuous outcomes; summary relative risk (RR) and 95% CI for the highest vs. lowest categories of coffee intake were estimated for binary outcome. For BMI, WMD was −0.08 (95% CI −0.14, −0.02); RR was 1.49 (95% CI 0.97, 2.29). For WC, WMD was −0.27 (95% CI −0.51, −0.02) and RR was 1.07 (95% CI 0.84, 1.36). In subgroup analysis by sex, evidence for an inverse association was more evident in men, specifically for continuous outcome, with WMD −0.05 (95% CI −0.09, −0.02) for BMI and −0.21 (95% CI −0.35, −0.08) for WC. Our meta-analysis suggests that higher coffee intake might be modestly associated with reduced adiposity, particularly in men.

## 1. Introduction

Having gained significant popularity worldwide in the last few decades, coffee has been a preferred source of caffeine for many people. Coffee is high in not only caffeine but also other bioactive compounds, such as polyphenol and chlorogenic acid that have been suggested to confer diverse health benefits [1]. Recent umbrella reviews concluded that higher coffee intake may be associated with decreased risk of type 2 diabetes, cardiovascular disease, certain cancers (e.g., breast, colorectal, endometrial, and prostate cancers), Parkinson’s disease, and mortality [2,3].

Considering that obesity has been established as a major underlying cause for the aforementioned health risks, it is plausible that coffee intake may be associated with reduced risk of obesity. Several studies have examined the effect of coffee intake on adiposity, as assessed by diverse anthropometric measures including body mass index (BMI) or waist circumference (WC) [4,5,6,7,8,9,10,11,12,13,14,15]. However, the results are largely inconsistent with results ranging from suggesting anti-obesity benefit [1,16,17] to reporting no effect [8,11] and even indicating increased obesity associated with higher coffee consumption [6,7]. With obesity reaching global epidemic proportions [18], it is critical to evaluate the current evidence on the effect of coffee intake on adiposity. Thus, we conducted a meta-analysis to systematically summarize the relationship between coffee intake and adiposity. 

## 2. Methods

The design, analysis, and reporting of this meta-analysis were based on the Meta-analysis Of Observational Studies in Epidemiology (MOOSE) checklist [19]. Three authors (AL, WL, SK) participated in literature search, study selection, and data extraction independently. Inconsistency between researchers was resolved through discussion with a fourth author (NK).

### 2.1. Search Strategy

PubMed and Embase were searched for epidemiologic studies published up to February 2019 using the provided search terms (Table S1). The language was restricted to English. Nonoriginal studies (e.g., case reports, commentaries, letters, and editorials) and animal experiments were excluded. The reference lists of all the articles included in this meta-analysis were reviewed for additional articles.

### 2.2. Study Selection

**Figure 1:**
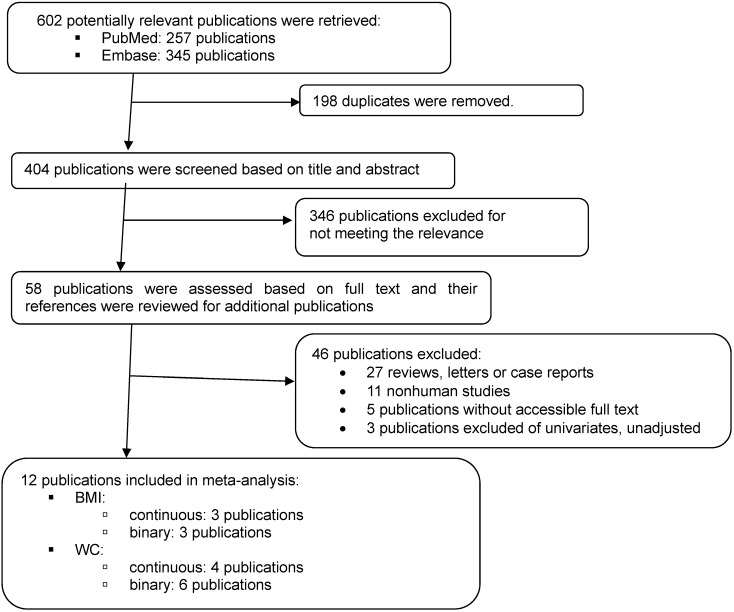
Flowchart for study selection.

To be included under the scope of this paper, studies had to be an observational study (e.g., cross-sectional, case-control, and cohort studies) or experimental study (e.g., randomized controlled trials) that examined the relationship between coffee intake and adiposity as indicated by weight, BMI, or WC. Studies that tested the effect of coffee extract supplements were excluded. We also excluded studies that reported results only from univariable analyses because estimates from these studies are highly likely to be confounded by factors such as age, alcohol intake, and smoking. Because obesity patterns in men and women generally differ, with men being more prone to abdominal adiposity (“apple shape”) and women more prone to lower-body adiposity (“pear shape”), coffee intake, if plays a role in inducing obesity, may exert differential effects by sex. For this reason, we primarily extracted separate estimates by sex unless studies provided only a pooled estimate. The flow diagram of the study selection is presented in Figure 1.

From each study, the following information was extracted: First author, publication year, study design, characteristics of study population (e.g., cohort name, country, age at enrollment, sex, sample size), unit and type of coffee intake, measure and definition of obesity, and variables controlled for. For studies that used continuous outcome (i.e., BMI, WC), mean or mean difference and corresponding standard deviation were extracted. For studies that used binary outcome (i.e., the status of overall and central obesity as indicated by BMI and WC, respectively), relative risk (RR) (odds ratio, rate ratio, or hazard ratio) and 95% confidence interval (CI) were extracted.

### 2.3. Statistical Analyses

To obtain summary estimates accounting for potential clinical and methodological variations across studies, we pooled the results using DerSimonian–Laird random effects model [20]. For continuous outcome, weighted mean difference (WMD) and 95% CI were obtained; for binary outcome, summary RR and 95% CI were estimated.

Heterogeneity in the relationship between coffee intake and adiposity across studies was quantified by I^2^ statistic, which represents the percentage of total variation across studies that is attributable to between-study heterogeneity [21]. I^2^ values of <25%, 25–49%, 50–74%, and ≥75% were considered to represent low, moderate, high, and very high heterogeneity, respectively. Potential for small study effects, such as publication bias, was assessed using Egger’s test [22]. Given a small number of studies, Egger’s test might have limited power to detect the bias. To address this issue, we considered there is evidence of small study effects if P_Egger_ < 0.1 instead of 0.05.

Subgroup analyses and meta-regression^9^ were conducted by *a priori* selected variables related to potential effect modifiers (e.g., sex).

For statistical significance, two-sided α was set at 0.05. All statistical analyses were conducted using STATA/SE V15.0 (StataCorp, College Station, TX, USA).

## 3. Results

### 3.1. Characteristics of Studies Included in the Meta-Analysis

A total of 12 studies were included in this meta-analysis [4,5,6,7,8,9,10,11,12,13,14,15]. Detailed information on these studies is delineated in Table S2. In brief, out of the 12 studies, six studies provided the results for overall adiposity based on BMI [5,6,7,8,11,13] and 10 studies for central adiposity based on WC [4,6,8,9,10,11,12,13,14,15]. By study design, 11 studies were cross-sectional studies and one study was a cohort study [15]. By country, five studies were conducted in Asia [6,10,12,13,14], five studies in Europe [4,7,9,11,15], and two studies in America [5,8]. Across the studies, excess coffee intake in the highest category compared to the lowest categories ranged from 0 cup/day [6,7,8,9] to >6 cups/day [15].

#### 3.1.1. For Overall Adiposity

##### Coffee Intake and Continuous BMI

**Figure 2:**
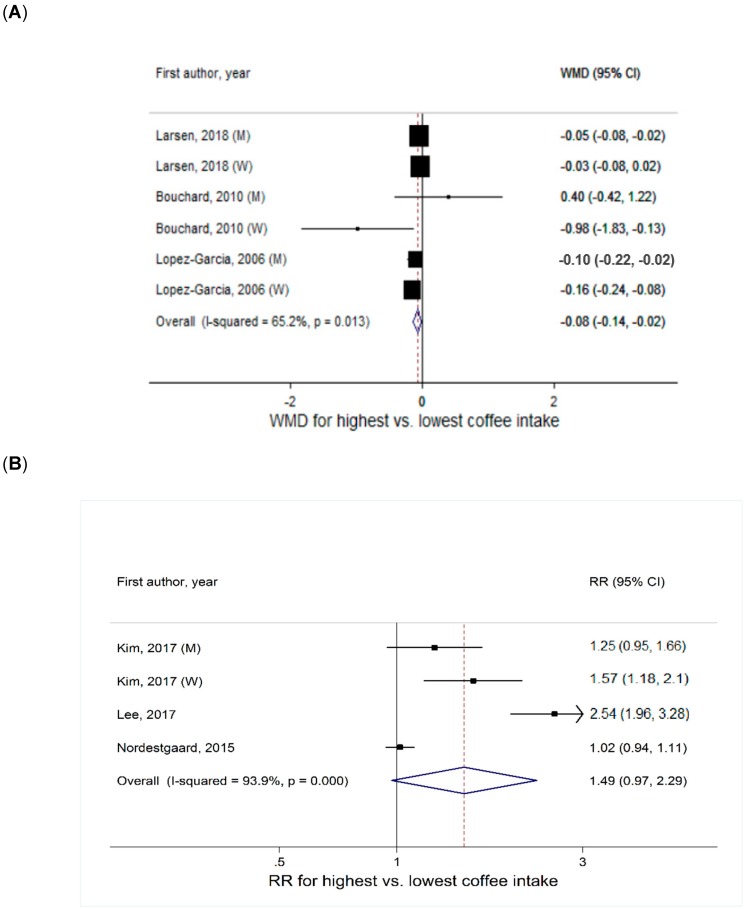
Meta-analysis of coffee intake with (**A**) continuous BMI and (**B**) overall obesity defined by BMI. Abbreviations: BMI, body mass index; CI, confidence interval; M, men; RR, relative risk; W, women; WMD, weighted mean difference.

Based on three studies [5,8,11] that reported outcomes in continuous BMI, the summary WMD in BMI comparing the highest vs. lowest categories of coffee intake was −0.08 (95% CI = −0.14, −0.02) (Figure 2A). While high heterogeneity was indicated (I^2^ = 65%), it was largely driven by difference in effect size and not by inconsistency in the direction of associations. There was no evidence of small study effects, such as publication bias (P_Egger_ = 0.39). By sex, a significant inverse association was found in men (WMD = −0.05, 95% CI = −0.09, −0.02, I^2^ = 0%) but not in women (WMD = −0.12, 95% CI = −0.27, 0.03, I^2^ = 84%) (Table 1). Heterogeneity by sex was not statistically significant (P_heterogeneity_ = 0.68).

##### Coffee Intake and Overweight or Obesity Defined by BMI

A total of three studies were included [6,7,13], of which two Asian studies [6,13] analyzed overweight and obesity as a single category. The summary RR of overweight or obesity comparing the highest vs. lowest categories of coffee intake was 1.49 (95% CI = 0.97, 2.29). There was very high heterogeneity (I^2^ = 94%) (Figure 2B), with studies conducted in South Korea [6,13] showing variable but strong positive associations (summary RR = 1.71, 95% CI = 1.12, 2.61, I^2^ = 86%). Small study effects, such as publication bias, were not indicated (P_Egger_ = 0.19). In subgroup analysis by sex, the association was not significantly heterogeneous (P_heterogeneity_ = 0.13), but a significant positive association was pronounced in women (summary RR = 2.01, 95% CI = 1.25, 3.21, I^2^ = 83%) than in men (summary RR = 1.25, 95% CI = 0.95, 1.65, I^2^ = not relevant) (Table 1).

#### 3.1.2. For Central Adiposity

##### Coffee Intake and Continuous WC

**Figure 3:**
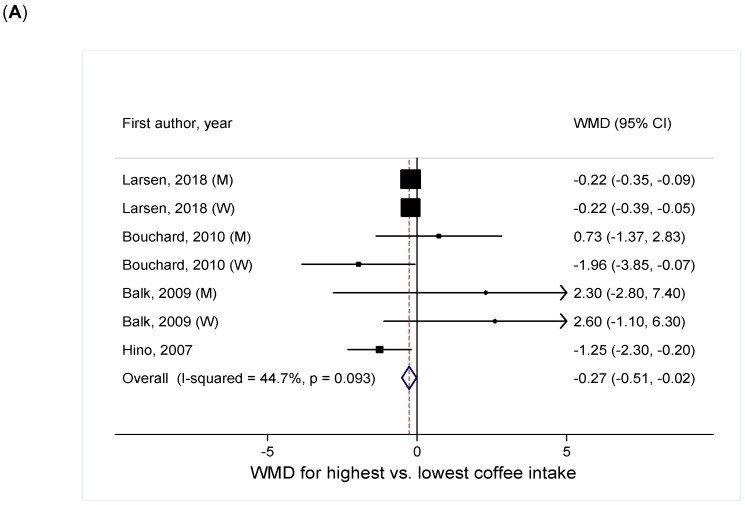

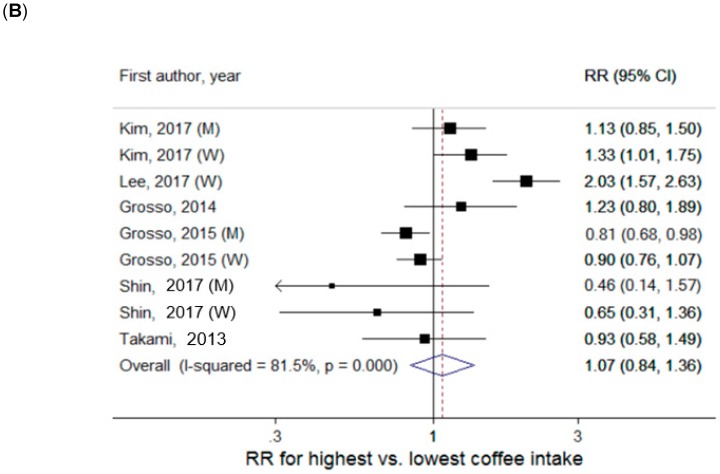
Meta-analysis of coffee intake with (**A**) continuous WC and (**B**) central obesity defined by WC. Abbreviations: CI, confidence interval; M, men; RR, relative risk; W, women; WC, waist circumference; WMD, weighted mean difference.

Based on four studies [8,10,11,15], the summary WMD in WC comparing the highest vs. lowest categories of coffee intake was −0.27 (95% CI = −0.51, −0.02), with moderate heterogeneity (I^2^ = 45%) (Figure 3A). There was no evidence of small study effects, such as publication bias (P_Egger_ = 0.98). Within each sex, an inverse association was suggested, but it was statistically significant only in men (WMD = −0.21, 95% CI = −0.35, −0.08, I^2^ = 0%) (Table 1). Heterogeneity by sex was not statistically significant (P_heterogeneity_ = 0.58).

##### Coffee Intake and Central Obesity Defined by WC

A total of six studies were included in this meta-analysis [4,6,9,12,13,14]. In defining central obesity, the cutoff of WC ≥ 90 cm was used in men except for one study (WC ≥ 85 cm) [12] and WC ≥ 80 cm was used in women except for three studies (WC ≥ 85 cm) [12,13,14]. The summary RR of central obesity comparing the highest vs. lowest categories of coffee intake was 1.07 (95% CI = 0.84, 1.36) (Figure 3B). The degree of heterogeneity in the associations was very high (I^2^ = 82%), with studies published in Asia showing opposite associations [6,12,13,14]. Small study effects were not indicated (P_Egger_ = 0.92). In subgroup analysis by sex, higher coffee intake was associated with reduced central obesity in men (summary RR = 0.90, 95% CI = 0.66, 1.23) but increased central obesity in women (summary RR = 1.18, 95% CI = 0.75, 1.86) (Table 1). However, the associations were not statistically significant and there was no evidence of heterogeneity by sex (P_heterogeneity_ = 0.59).

## 4. Discussion

In this meta-analysis of observational epidemiologic studies, higher coffee intake was significantly associated with modestly lower BMI and WC in men but not in women. However, these findings were not replicated in the meta-analysis of observational epidemiologic studies that defined overall or central obesity using specific cutoffs of BMI and WC. For overweight or obesity as defined by BMI, there was no evidence of an association in men, while a higher coffee intake was significantly associated with an increased risk in women. For central obesity as defined by WC, higher coffee intake was associated with reduced risk, albeit statistically nonsignificant, in men; a statistically nonsignificant but positive association was indicated in women.

For BMI and WC, respectively, the inconsistent findings between the continuous and binary outcomes can be explained from several perspectives. First, because no study examined coffee intake in relation to both continuous and binary anthropometric measures, different studies contributed to our meta-analyses of continuous and binary forms of a given adiposity measure. Studies differ in diverse factors including study design, population, and methodological quality, and these variations may have contributed to the inconsistency. For instance, studies conducted in Korea where instant coffee mixes containing sugar and creamer account for a major proportion of overall coffee consumption were included in the meta-analyses of binary outcome only, mostly contributing highly positive associations between coffee intake and obesity [6,13]. Alternatively, the discrepant findings may represent a minimal benefit of coffee intake in controlling adiposity. In case of coffee intake and WC in men, the lack of a significant inverse association with the status of central obesity may suggest that the magnitude of association between coffee intake and continuous WC is not substantial enough for extra cups of coffee per day to prevent the progression from normal status to central obesity.

Despite the modest degree of association between coffee intake and continuous BMI and WC, there are several biological mechanisms that add support to the anti-obesity benefit of coffee. Biologically active compounds in coffee, such as chlorogenic acid, caffeine, trigonelline, and magnesium, have shown to be associated with anti-obesity benefits [1]. Supplementation with chlorogenic acid reduced body weight, visceral fat mass, and triglyceride content in adipose tissue in high-fat-fed mice [23]. In an in vitro study, trigonelline inhibited adipocyte proliferation and lipid accumulation in differentiating adipocytes [24]. A pronounced benefit of coffee among men than among women may be in part related to greater susceptibility of men toward obesity, particularly visceral obesity, originating from genetic variations [25] and sex hormones [26].

There are several limitations in our meta-analysis. First, our study extends the limitations of the included studies, which were mostly cross-sectional. Thus, the validity of our meta-analysis to infer temporal relationship between coffee intake and adiposity is compromised; residual confounding by smoking cannot be ruled out. Larson et al. performed a sensitivity analysis among never smokers and an association became attenuated and statistically nonsignificant [11]. Additionally, due to insufficient data, no meta-analysis was conducted by type of coffee (e.g., caffeinated vs. decaffeinated, sweetened vs. unsweetened, added cream vs. no cream, etc.), which may have differential effects on adiposity. It is notable in our meta-analysis on central obesity that most studies published in Korea, where instant coffee mixes containing sugar and cream are widely consumed, found a positive association [6,13]. In the Lee study [6], a positive association was found even after the adjustment for sugar and cream additive use as covariates in their multivariable regression models. On the contrary, in another study conducted in Korea that defined coffee consumption as drinking coffee without sugar and cream by selecting subjects who responded that they consumed coffee with “almost no sugar added” and “almost no cream added,” an inverse association, albeit not statistically significant, was observed [14]. Furthermore, the highest vs. lowest categories of coffee intake ranged from 0 cup/day to >6 cups/day among the studies included in our meta-analysis. However, we did not conduct a separate meta-analysis based on the difference in coffee intake levels between the extreme categories because only a few studies provided all required information for such analysis. Only three studies [6,7,15] provided an estimate for at least 3 cups/day of difference in intake and they reported discordant endpoints (continuous BMI, binary BMI, continuous WC, binary WC). Finally, our meta-analysis included a small number of studies. However, based on Egger’s test, we found no evidence of small study effects, such as publication bias, in our results even after using more strict cutpoint (P_Egger_ < 0.1). Although Egger’s test can be low-powered given a small number of studies, Egger’s test is still more sensitive in detecting many types of biases associated with small studies compared with other equivalent tests such as Begg’s test.

Yet, our meta-analyses have strengths as well. To our knowledge, this is the first meta-analysis that summarized the relationship between coffee intake and adiposity. By analyzing coffee intake in relation to both BMI and WC, and in both continuous and binary forms, this meta-analysis exhaustively examined any potential effect that coffee intake may have on both the amount and the distribution of adiposity. As a meta-analysis combines multiple studies, the increased statistical power enabled us to detect a modest benefit of coffee intake in reducing adiposity, as indicated by a small reduction in BMI and WC.

In conclusion, coffee intake may be modestly associated with lower adiposity as indicated by BMI and WC, particularly in men. While the benefit of coffee intake against obesity was not as fundamental as balanced diets and physical activity, in view of considerable evidence supporting the protective effect of coffee against major chronic diseases [2,3], coffee may be incorporated into a part of healthy lifestyle to promote overall health.

## Figures and Tables

**Table 1:** Meta-analyses of coffee intake and overall and central adiposity by sex.

Outcome	Among Men	Among Women	P_heterogeneity_ by Sex
**Overall adiposity**			
**BMI**			
No. of studies	3	3	
WMD (95% CI; I^2^)	−0.05 (−0.09, −0.02; 0%)	−0.12 (−0.27, 0.03; 84%)	0.68
**Overweight or obesity as defined by BMI**			
No. of studies	1	2	
RR (95% CI; I^2^)	1.25 (0.95, 1.65; NR)	2.01 (1.25, 3.21; 83%)	0.13
**Central adiposity**			
**WC**			
No. of studies	3	3	
WMD (95% CI; I^2^)	−0.21 (−0.35, −0.08; 0%)	−0.36 (−2.00, 1.28; 64%)	0.58
**Central obesity as defined by WC**			
No. of studies	3	4	
RR (95% CI; I^2^)	0.90 (0.66, 1.23; 59%)	1.18 (0.75, 1.86; 90%)	0.59

Abbreviations: BMI, body mass index; CI, confidence interval; NR, not relevant; RR, relative risk; WC, waist circumference; WMD, weighted mean difference.

## Supplementary Materials

The following are available online at https://www.mdpi.com/2072-6643/11/6/1274/s1, Table S1: Database search strategy, Table S2: Characteristics of studies included.
