# Supplementary material for: Coffee Intake and Obesity: A Meta-Analysis
Source: Nutrients. 2019 Jun 5;11(6):1274. doi: 10.3390/nu11061274 (PMC6628169; doi:10.3390/nu11061274)
Supplement: Supplementary file 1 [file nutrients-11-01274-s001.pdf]

Supplementary table 1. Database search strategy

**PubMed**	("Coffee"[Mesh] OR "Caffeine"[Mesh] OR coffee[tw] OR caffeine[tw] OR decaffeinated[tw] OR fluid intake[tw] OR tea[tw]) AND ("Body Weight"[Mesh] OR weight[tw] OR weight loss*[tw] OR weight gain*[tw] OR weight change*[tw] OR anthropometry[tw] OR birth weight[tw] OR birthweight[tw] OR birth-weight[tw] OR obesity[tw] OR obese[tw] OR overweight[tw] OR over-weight[tw] OR body composition[tw] OR body mass index[tw] OR BMI[tw] OR fat mass[tw] muscle mass[tw] OR waist circumference[tw] OR fat distribution[tw] OR hip circumference[tw] OR waist hip ratio*[tw] OR skinfold measurement*[tw] OR skinfold thickness[tw] OR DEXA[tw] OR bio-impedence[tw]) NOT (Case Reports[ptyp] OR Comment[ptyp] OR Letter[ptyp] OR Editorial[ptyp]) NOT ("animals" [Mesh] NOT "humans" [Mesh])
**Embase**	("Coffee"/exp OR "Caffeine"/exp OR coffee:ti,ab OR caffeine:ti,ab OR decaffeinated:ti,ab OR fluid intake:ti,ab OR tea:ti,ab) AND ("Body Weight"/exp OR "obesity"/exp OR weight:ti,ab OR 'weight loss*':ti,ab OR 'weight gain*':ti,ab OR 'weight change*':ti,ab OR anthropometry:ti,ab OR 'birth weight':ti,ab OR birthweight:ti,ab OR birth-weight:ti,ab OR obesity:ti,ab OR obese:ti,ab OR overweight:ti,ab OR over-weight:ti,ab OR 'body composition':ti,ab OR 'body mass index':ti,ab OR BMI:ti,ab OR 'fat mass':ti,ab 'muscle mass':ti,ab OR 'waist circumference':ti,ab OR 'fat distribution':ti,ab OR 'hip circumference':ti,ab OR 'waist hip ratio*':ti,ab OR 'skinfold measurement*':ti,ab OR 'skinfold thickness':ti,ab OR DEXA:ti,ab OR bio-impedence:ti,ab) NOT ('case report'/de OR 'editorial'/de OR 'letter'/de OR 'review'/de) NOT ([animals]/lim NOT [humans]/lim)

Supplementary table 2. Characteristics of studies included

First author, Year, Country, Reference	Cohort name, Study period	Analysis Design	Sex, Age at baseline (years)	Outcome definition	Diet assessment method (validation)	Highest vs lowest coffee intake (cups/day)	RR (95% CI) or Mean difference (95% CI)	Variables adjusted for
Larsen 2018 Denmark	MONICA Study	Cross-sectional analysis	M 46	Continuous BMI	FFQ	per 1 cup/day	-0.05 (-0.09, -0.02)	age, sex, total energy intake, current smoking, and habitual physical activity, smoking status, physical activity, school education, gender, menopausal status for women, height
				Continuous WC		per 1 cup/day	-0.22 (-0.35, -0.09)	
			W 46	Continuous BMI		per 1 cup/day	-0.03 (-0.08, 0.02)	
				Continuous WC		per 1 cup/day	-0.22 (-0.39, -0.05)	

Kim<br>2017<br>South Korea	KNHANES<br>2009 - 2010	Cross-sectional analysis	M<br>≤ 40	Binary BMI<br>(overall obesity: ≥25)	FFQ	≥ 3 vs. < 1	1.25<br>(0.95, 1.66)	age, smoking, alcohol intake, macronutrient intake, exercise, family income
				Binary WC<br>(central obesity: ≥90cm)		≥ 3 vs. < 1	1.13<br>(0.85, 1.50)	
			W<br>≤ 40	Binary BMI<br>(overall obesity: ≥25)		≥ 3 vs. < 1	1.57<br>(1.18, 2.10)	
				Binary WC<br>(central obesity: ≥85cm)		≥ 3 vs. < 1	1.33<br>(1.01, 1.75)	
Lee<br>2017<br>South Korea	KNCC<br>2007 - 2016	Cross-sectional analysis	W<br>30 - 70	Binary BMI<br>(overall obesity: ≥25)	FFQ	≥ 3 vs. 0	2.54<br>(1.96, 3.28)	age, level, occupation, alcohol, smoking status, regular exercise, total calorie intake, use of the sugar and creamer additives
				Binary WC<br>(central obesity: ≥80cm)		≥ 3 vs. 0	2.03<br>(1.57, 2.63)	
Shin<br>2017<br>South Korea	KNHANES<br>2013 - 2014	Cross-sectional analysis	M<br>20 ≤	Binary WC<br>(central obesity: > 90cm)	FFQ	≥ 3 vs. < 1	0.46<br>(0.14, 1.57)	age, BMI, household income, education, alcohol, smoking, exercise, daily caloric intake.
			W<br>20 ≤	Binary WC<br>(central obesity: > 85cm)		≥ 3 vs. < 1	0.65<br>(0.31, 1.36)	
Nordestgaard<br>2015<br>Denmark	CGPS, CCHS<br>1991 - 1994<br>2001 - 2003	Cross-sectional analysis	M + W<br>42 - 71	Binary BMI<br>(overall obesity: >30)	Questionnaires	> 5 vs. 0	1.02<br>(0.94, 1.11)	age, sex, smoking, physical activity, antihypertensive and lipid-lowering medication to estimate associations of

								coffee intake with BMI, WC, weight, height, systolic and diastolic blood pressure, plasma triglycerides, high-density lipoprotein cholesterol and plasma glucose
Grosso 2014 Italy	Sicily mediterranean study 2009 - 2010	Cross-sectional analysis	M + W 50	Binary WC (central obesity: WC ≥90cm in men and WC≥ 80cm in women)	FFQ	> 2 vs. 0	1.23 (0.80, 1.89)	age, gender, energy intake, adherence to the Mediterranean diet (MedDietScore), smoking habit and physical activity
Grosso 2015 Poland	HAIPEE 2002 - 2005	Cross-sectional analysis	M 45 - 69	Binary WC (central obesity: ≥90cm)	FFQ	> 2 vs. < 1	0.81 (0.68, 0.98)	age, energy intake, educational level, occupational level, alcohol consumption, tea consumption, smoking habit and physical activity
			W 45 - 69	Binary WC (central obesity: ≥ 80cm)		> 2 vs. < 1	0.90 (0.76, 1.07)	
Takami 2013 Japan	J-MICC	Cross-sectional analysis	M + W 35 - 70	Binary WC (central obesity: WC ≥90cm in men and WC≥ 85cm in women)	FFQ	≥ 3 vs. < 1.5	0.93 (0.58, 1.49)	age, sex, total energy intake, physical activity, smoking and drinking habits

Bouchard<br>2010<br>USA	NHANES	Cross-sectional analysis	M<br>18 ≤	Continuous BMI	Questionnaires	≥2 vs. 0	0.40<br>(-0.42, 1.22)	age, gender, ethnicity, smoking, alcohol, physical activity, diet quality index, total calorie intake, and the frequency of coffee and tea consumption.
				Continuous WC		≥2 vs. 0	0.73<br>(-1.37, 2.83)	
			W<br>18 ≤	Continuous BMI		≥2 vs. 0	-0.98<br>(-1.83, -0.13)	
				Continuous WC		≥2 vs. 0	-1.96<br>(-3.85, -0.07)	
Balk<br>2009<br>Netherland	AGAHLS	Cohort analysis	M<br>27 - 42	Continuous WC	Questionnaires	>6 vs. ≤2	2.30<br>(-2.80, 7.40)	energy intake, physical activity, smoking and alcohol consumption
			W<br>27 - 42	Continuous WC		>6 vs. ≤2	2.60<br>(-1.10, 6.30)	
Hino<br>2007<br>Japan	Tanushimaru Seven country studies	Cross-sectional analysis	M + W<br>40 ≤	Continuous WC	FFQ	Increased vs. Decreased	-1.25<br>(-2.30, -0.20)	age, sex, total energy intake, alcohol intake, current smoking, habitual physical activity

Lopez-Garcia 2006	NHS 1986 – 1998	Cross-sectional Analysis	M 40-75	Continuous BMI	FFQ	Increased vs. Decreased	-0.10 (-0.22, -0.02)	age, BMI at baseline, smoking status, physical activity, alcohol intake (quintiles), quintiles of baseline and change in trans-fat intake, glycemic load, total fiber and whole-grain intakes, and quartiles of baseline and change in low- and high-calorie soft drink intake, and fruit and vegetable consumption
			W 30-55	Continuous BMI		Increased vs. Decreased	-0.16 (-0.24, -0.08)	

Abbreviations

AGAHLS: Amsterdam Growth and Health Longitudinal Study, BMI: Body Mass Index, CGPS: Copenhagen General Population Study, FFQ: Food Frequency Questionnaire, HAPIEE: Health, Alcohol and Physiological factors In Eastern Europe, J-MICC: The Japan Multi Institutional Cohort Study, KNCC: Korean National Cohort Study, KNHANES: The Korea National Health and Nutrition Examination Survey, NHANES: National Health and Nutrition Examination Survey, NHS: National Health Service

No.: Number, OR: Odds Ratio, RR: Risk Ratio, SD: Standard Deviation, WC: Waist Circumference
